# SENP1 is required for the growth, migration, and survival of human adipose-derived stem cells

**DOI:** 10.1080/21623945.2020.1863625

**Published:** 2020-12-29

**Authors:** Yingying Wu, Beixin Yu, Min Wang

**Affiliations:** Center for Translational Medicine, The First Affiliated Hospital, Sun Yat-sen University, Guangzhou, China

**Keywords:** Human adipose-derived stem cells, SENP1, proliferation, migration, apoptosis

## Abstract

Human adipose-derived stem cells (hADSCs) are adult mesenchymal cells that have attracted the interest of clinical scientists and surgeons due to their large number of advantages including ease of access and expansion, abundance in cell culture, high proliferative rates, and lower senescence. SUMO/sentrin specific protease 1 (SENP1) is a critical protease that is required during the process of SUMOylation and deSUMOylation, which are dynamic mechanisms that influence cell cycle progression, cell proliferation, and apoptotic status. However, the contribution of SENP1 to these important cellular processes in hADSCs is largely uncharacterized and further studies in this area are required. Here, we show for the first time that after knock out SENP1 in hADSCs, their capacity to migrate and proliferate were inhibited, while apoptosis was enhanced. However, SENP1 did not significantly influence the morphology and MSC-related phenotypes of the hADSCs. These results highlight a role for SENP1 during hADSC growth, and its potential as a therapeutic target to improve the efficacy and safety of hADSCs in the clinic.

## 1. Introduction

Recently, stem cell-based therapies have emerged as a potential and dynamic option for tissue repair for which rapid progress has been achieved in both the preclinical and clinical setting. The application of stem cell therapy is becoming increasingly popular due to their self-renewal and differentiation capabilities. Stem cells can be categorized as embryonic (ESCs) or induced pluripotent stem cells (iPSCs) dependent on their site of origin. Mesenchymal stem cells (MSCs) originate from the mesodermal, and represent adult stem cells with multidirectional differentiation potential. MSCs can be harvested from bone marrow extraction [1], adipose tissue isolation [2], dental tissue [3], and other sources [4]. However, assays that can effectively identify and characterize MSCs are lacking [5], despite the advanced knowledge of their biological capabilities [6]. According to the International Society of Cellular Therapy, MSCs have several unique characteristics including their spindle-shape, plastic-adherence, unique antigen markers, and their potential to differentiate into adipocytes, osteocytes, and chondrocytes [7].

The use of adipose-derived stem cells (ADSCs) has attracted intense research interest.

In 2001, ADSCs were characterized as MSCs by Zuk and colleagues [8] and have since been shown to harbour many advantages over stem cells derived from other tissue origins. For example, ADSCs are easily obtained from adipose tissue by minimally invasive techniques with low morbidity and a lack of ethical concerns. Liposuction is one such example of a non-invasive source of these cells. ADSCs are also permissive to standard cell culture assessments in which they display high proliferative rates. Besides the advantage of abundance in cell culture, hADSCs have lower rates of cell senescence.

These years hADSCs have emerged as a novel source of stem cells during cell regeneration with various therapeutic effects through their capacity to enhance tissue regeneration following damage. Recently, reports have demonstrated that hADSCs may accelerate wound healing by promoting angiogenesis, modulating immune response, and epithelialization in the wound [9]. Furthermore, hADSCs are likely to treat autoimmune, inflammatory, and degenerative diseases because of their multilineage potential, secretion of anti-inflammatory molecules and immunoregulatory effects [10].

However, a drawback of hADSC usage is their low rates of donor cell engraftment and survival. Knowledge of the mechanisms and functions of hADSCs are therefore crucial to the development of methods that can enhance cell survival and retention, so as to promote therapeutic efficacy. Such advances would add to the intense interest in these cells from clinical scientists and surgeons alike.

As a key post-translational modification, SUMOylation regulates a multitude of physiological and biological processes in mammalian cells. SUMOylation is regulated by a multistep enzymatic cascade that facilitates the attachment of SUMO molecules to substrates, which can be removed by Sentrin/SUMO-specific proteases (SENPs) through deSUMOylation [11]. Recent studies have demonstrated that SUMOylation stimulates survival and neurogenesis of neuronal stem cells in the aetiology of neurodegenerative disorders [12]. In some reports, SUMOylation is closely related to the development of liver disease and a variety of cancers such as breast cancer [13,14]. Nevertheless, as a critical signalling pathway in cancer stem cells, SUMOylation is considered as potential therapeutic targets in cancer [15,16].

In the processes of SUMOylation and deSUMOylation, the small ubiquitin-like modifier (SUMO) and its specific proteases (SENPs) are essential signal molecules. Reports showed that they might be novel mitochondrial stress sensors that respond to the signals under different types of stresses [17]. SENP1 and SENP2 have been applied to discover new biomarkers for diagnosis or treatment for breast cancer [14].

SENP1 (Sentrin-specific protease 1) is a protease that is essential for deSUMOylation [11]. In recent years, SENP1 has been shown to play an essential role in cellular apoptosis and proliferation [18,19]. Furthermore, we recently demonstrated that SENP1 knockout mice develop glucose-related disorders [20], and we also found that during graft arteriosclerosis, SENP1-mediated GATA2 deSUMOylation plays critical role in promoting endothelial activation [21].

Previous studies highlight the involvement of SENP1 in adipocytes and cancer cells, but the specific functions of SENP1 in human ADSCs remain poorly defined. Herein, we describe new roles for SENP1 in ADSCs that provide more detailed information on the role of SENP1 in cell survival and highlight its role during hADSC growth, and its potential as a therapeutic target to improve the efficacy and safety of hADSCs in the clinic.

## 2. Result

### 2.1. Characterization and identification of hADSCs

**Figure 1.**
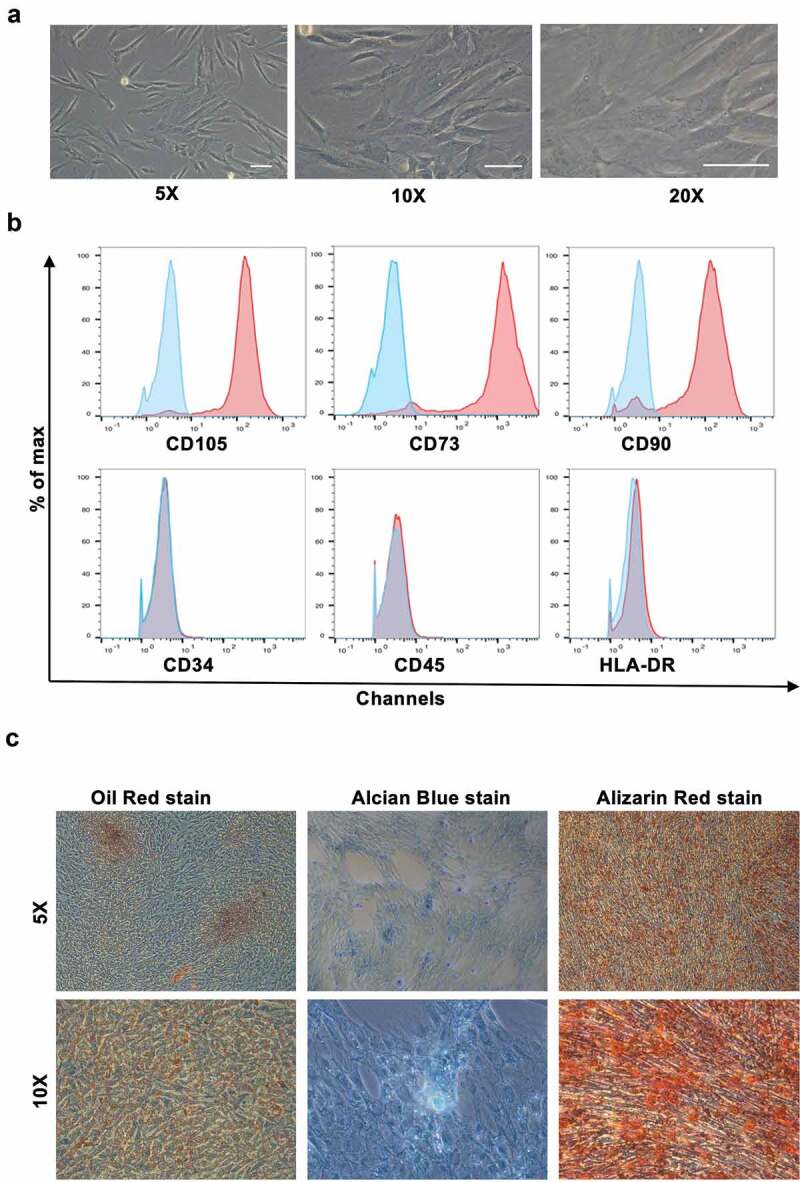

Adipose tissues were obtained from patients receiving liposuction at our institute. Samples were ground and digested in Collagenase Type I and resuspended in Mesenchymal Stem Cell Basal Medium (MSCBM). Typical samples consisted of mature adipocytes and a heterogeneous stromal vascular fraction (SVF) that included various cell types [22]. The hADSCs were digested with collagenase and centrifuged on a density gradient for their isolation [23]. From a morphological perspective, the hADSCs were fibroblast-like and grew in adherent monolayers. The hADSCs also lacked the large intracellular lipid droplets typically observed in adipocytes (Figure 1(a)). The hADSCs expressed CD73, CD90, and CD105 at their cell surface, but lacked HLA, CD45, and CD34 as assessed through flow cytometry (Figure 1(b)). Moreover, they were identified to have capacities of self-renew and multilineage differentiation. When hADSCs were cultured in appropriate induction medium, they differentiated into adipocytes (Oil Red positive), chondrocytes (Alcian Blue positive), and osteocytes (Alizarin red positive) (Figure 1(c)). Accordingly, the hADSCs were isolated successfully for they possessed the typical characteristics of MSCs.

### 2.2. SENP1 silencing in hADSCs

**Figure 2.**
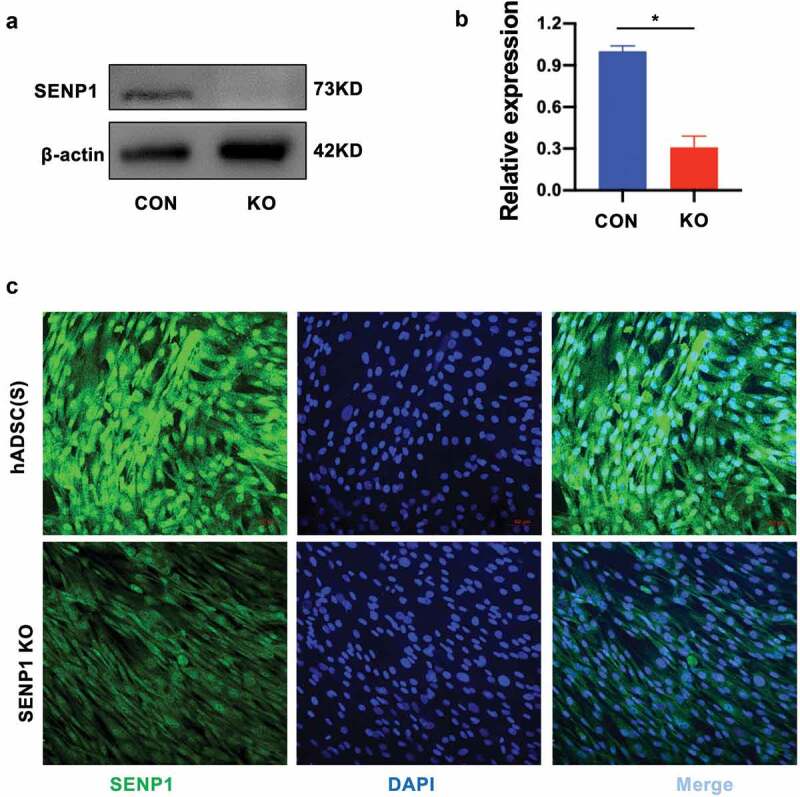

In order to investigate the influence and functions of SENP1 in hADSCs, we used CRISPR-Cas9 to knockout SENP1 in hADSCs. sgRNAs were cloned into lentiCRISPRv2 (ACATGCCATACTTCCGGAAGCGG) and targeting vectors containing two expression cassettes were constructed that contained the hSpCas9 and chimeric guide RNA. Cells expressing the SENP1 knockdown system showed lower levels of SENP1 expression as highlighted by western blot analysis using anti-SENP1 antibodies (Figure 2(a–b)). Furthermore, through immunofluorescence assays, SENP1-silenced cells showed lower levels of nuclear staining when compared to scrambled controlled cells (Figure 2(c)). Taken together, this highlighted our ability to perform SENP1 knockouts in hADSCs to investigate its functionality in these cells.

### 2.3. Effects of SENP1 knockout on the morphology and surface markers of hADSCs

**Figure 3.**
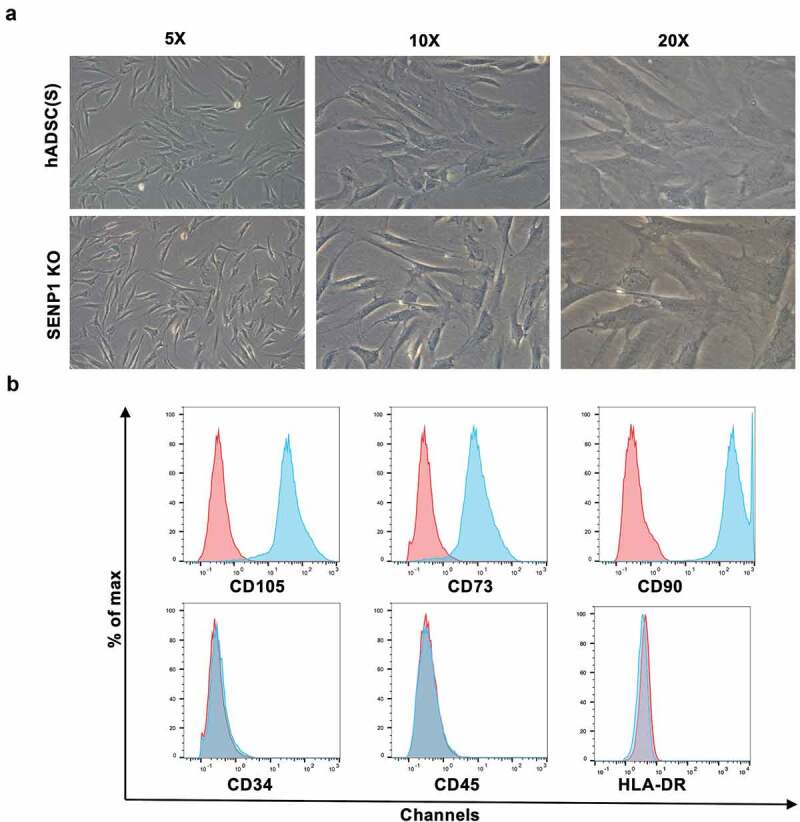

To examine whether the knockout of SENP1 in hADSCs leads to changes in their morphology and MSC-related phenotypes, we observed the morphology of both hADSCs and hADSCs after knockout SENP1. Surprisingly, no changes in adherent ability and cell shape were observed; both cell types adhered to the plastic and presented a fibroblast-like growth pattern (Figure 3(a)). Through flow cytometry assays, both cell types were positive for CD73, CD90, CD105, but negative for CD34, CD45, and HLA-DR (Figure 3(b)).

### 2.4. Effects of SENP1 knockout on the migration and proliferation of hADSCs

**Figure 4.**
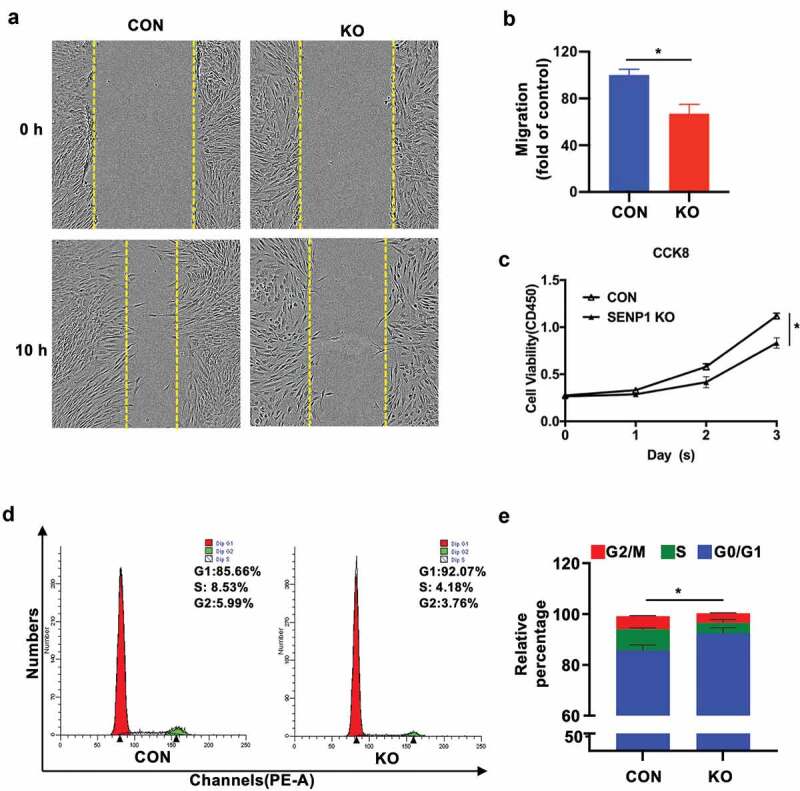

Scratch wound healing test were performed to detect the effects of SENP1 on cell migration. We found that after 10 h, SENP1-silenced hADSCs moved more slowly than the control hADSCs (Figure 4(a–b)). To further demonstrate the effects of SENP1 on cell proliferation, CCK-8 assays were performed, revealing a growth defect in knockout cells (Figure 4(c)). We next assessed the effects of SENP1 knockout on cell cycle progression through flow cytometry analysis. We found that SENP1 deletion led to a higher proportion of cells in the G0/G1 phase compared to the control group (Figure 4(d–e)). These results indicate that the deletion of SENP1 in hADSCs leads to a loss of cell migration, and inhibits cell proliferation through inducing cell cycle arrest during G0/G1 to S phase transition.

### 2.5. Effects of SENP1 knockout on the apoptotic status of hADSCs

**Figure 5.**
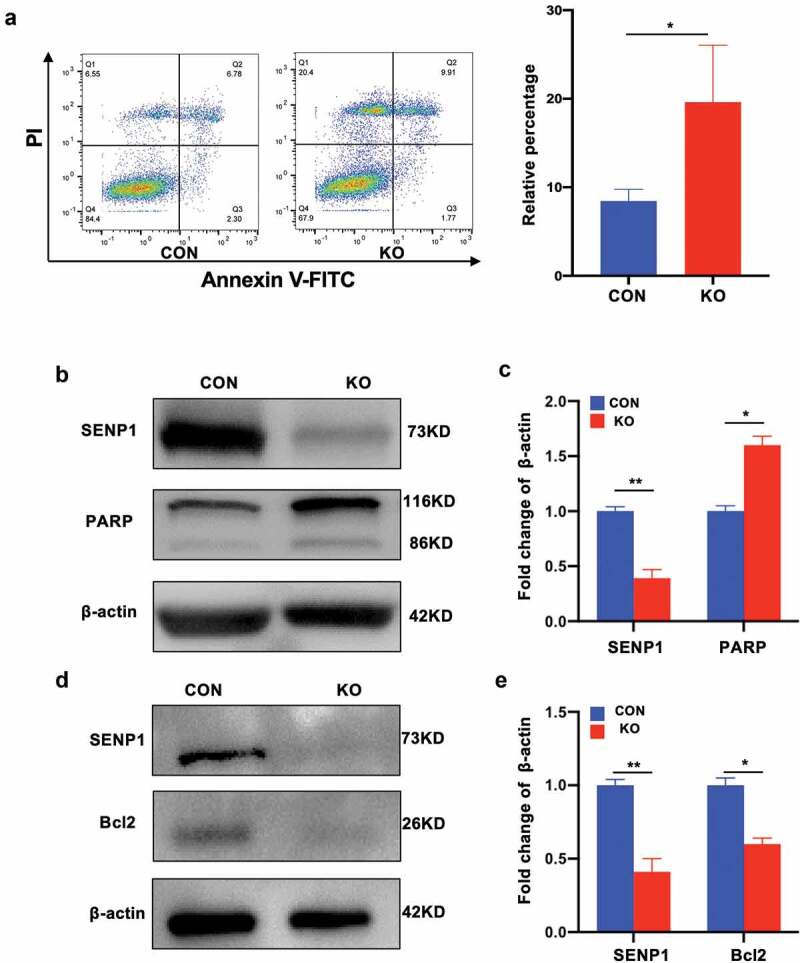

We further investigated whether SENP1 knockout influenced hADSC apoptosis through flow cytometry and western blot analysis. For these experiments, apoptotic rates were measured using commercially available Annexin V/propidium iodide (PI) kits. We found that cell surface antigens and phenotypes related to apoptosis were more pronounced in hADSCs following SENP1 silencing (Figure 5(a)), and the expression of pro-apoptosis like cleaved PARP increased (Figure 5(b–c)) whilst anti-apoptosis markers such as Bcl2 were downregulated (Figure 5(d–e)). These data suggest that SENP1 silencing promotes apoptosis in hADSCs.

## 3. Materials and Methods

### 3.1. Isolation and expansion of hADSCs

Adipose tissues were obtained from patients receiving liposuction at our institute. Samples were ground and digested in Collagenase Type I (Gibco, Invitrogen) for 60 min at 37°C with shaking. After digestion, cell suspensions were filtered (75-μm) to remove solid aggregates. Samples were then centrifuged (5 min at 1500 rpm on 3 occasions) to produce a pure stromal vascular cell fraction. Cells were then washed and resuspended in Mesenchymal Stem Cell Basal Medium (MSCBM; DAKEWEI) supplemented with 5% UltraGRO-Advanced (GMP Grade, Helios), 100 U/mL penicillin, and 100 mg/mL streptomycin, and were cultured at standard temperature and CO2 conditions. After 3 d, cells were washed to detach non-adherent cells and then were supplemented with fresh MSCBM every 2 d thereafter. Cells were grown to ~80% confluency for all experiments and morphological assessments performed on an inverted light microscope. Cells as passage 3 cells were used for all subsequent assays.

### 3.2. Multilineage potential and characterization

To assess the ability of the hADSCs to undergo adipogenic differentiation, cells (2 × 10^4) were seeded into 6-well plates in complete MSCBM media and grown to 100% confluency. Cells were then treated with adipogenic differentiation medium (HUXMD-90,031, Cyagen) every 3 d for a 21 d period. Cells were then washed, fixed in 4% formaldehyde and stained with oil red O. For the assessment of osteogenic differentiation, cells were induced with osteogenic differentiation medium (GUXMX- 90,021, Cyagen) for 21 d, prior to fixation and Alizarin Red S staining. Cells were then imaged and osteogenic differentiation was assessed. Positive cells presented as nacarats.

Chondrogenic differentiation was achieved through culturing 2.5 × 105 cells into 15 mL polypropylene culture tubes containing chondrogenic differentiation medium (GUXMX-90,041, Cyagen). Briefly, gas exchange was permitted by loosening the screw caps containing the cells and tubes were incubated at 37°C in 5% CO2. Cells were replenished with fresh media every 2–3 d and cultured for a total of 20 d. Cell pellets were then formalin-fixed and paraffin-embedded for alcian blue stain analysis. Cells were imaged on an optical microscope.

For flow cytometry 5 × 10^5 ADSCs (in 100 μL PBS) were incubated with fluorescently labelled monoclonal antibodies (CD34-FITC (BioLegend), CD45-PE-CY5 (BD Pharmingen), CD73-PE (BioLegend), CD90-PE-CY5 (BioLegend), CD105-PE (eBioscience) and HLA-DR-FITC (BioLegend)) in the dark at 2–8°C for 30 min. Cells were then washed in PBSM and the fluorescence of the hADSCs quantified.

### 3.3. SENP1 silencing through CRISPR/Cas9

SENP1 knockdowns were performed in hADSCs using the CRISPR/Cas9 system. sgRNAs were designed (http://chopchop.cbu.uib.no/index.php), and then cloned into lentiCRISPRv2. The sequence of the sgRNA of SENP1 was ACATGCCATACTTCCGGAAGCGG. Next, a targeting vector containing two expression cassettes was constructed that contained the hSpCas9 and chimeric guide RNA. The vector was digested with BsmBI and paired annealed oligos were cloned into the single guide RNA scaffold. The oligos were designed based on the target site sequence (20 bp) and were flanked on the 3ʹ end by a 3 bp NGG PAM sequence. We then co-transfected the pVSVG and psPAX2 packaging plasmids into HEK293T cells. CMV-EGFP lentiviral transfer plasmids were used as a positive control for transfection. Cell supernatants were then collected, filtered through a 20 μm micropore filter, and cultured in a 1:1 hADSC cells basal media for 48 h. hADSCs lacking SENP1 were selected from puromycin (1.5 μg/ml, Sigma) resistant clones.

### 3.4. CCK-8 assays

Cells viability was assessed through CCK-8 assays. Briefly, cells were plated into 96 wells (2500 cells/well) and were treated with 10 μl of CCK-8 solution (Dojindo, Tokyo, Japan) at 37°C for 2 h. OD values at 450 nm were then measured on a microplate reader (Thermo, Multiskan MK3).

### 3.5. Flow Cytometry for apoptosis assessments

Apoptotic rates were measured using commercially available Annexin V/propidium iodide (PI) kits (Nanjing KGI, China) which include solution A: FITC labelled Annexin-V; solution B: propidium iodide solution (PI); solution C: binding buffer. Cells were freshly prepared prior to labelling and resuspended in 500 μl of reaction containing 5 μl of solution A and 5 μl of solution B, samples were mixed and ~200,000 cells were taken from each tube and washed twice with PBS. Supernatants were then discarded and 500 μl Annexin-V working solution was added to the samples at room temperature for 25 mi. Cells (~10,000) were then assessed through flow cytometry.

### 3.6. Cell cycle analysis

To assess cell cycle status, cells were fixed in ice-cold ethanol (75%) and stored at −20°C overnight. Cells were then treated with Tris-HCl buffer (pH 7.4) supplemented with 100 µg/ml RNase A and subsequently stained with propidium iodide (PI; 50 µg/ml). Cells were analysed on a flow cytometer and cell cycle distribution was assessed through ModFit analysis.

### 3.7. Wound healing assay

Confluency cells monolayers plated into 96-well plates were subjected to a needle-induced scratch. Cells were then washed to remove cell debris and incubated in serum-free medium under standard culture conditions. Cells were imaged at 0 h, 10 h, and 24 h on an inverted microscope connected to a DXM1200 digital camera (Nikon, Tokyo, Japan).

### 3.8. Immunofluorescence analysis

For immunofluorescent assessments, cells were fixed with 4% PFA for 15 min and permeabilized in 0.1% Triton X-100 for 10 min. Cells were then washed in PBS (x3) and blocked in 5% BSA for 1 h. Cells were then probed with rabbit antibodies against SENP1 (Santa Cruz) overnight and labelled with fluorescently conjugated anti-rabbit IgG (Jackson Immunoresearch). Cell nuclei were then stained with DAPI and the nuclei visualized on a Leica DMI 6000 fluorescent microscope.

### 3.9. Western blot analysis

Adipocytes were lysed in 50 mM Tris-HCl, pH 7.4, 150 mM NaCl, 1 mM EDTA, 1% NP-40, and 0.1% sodium dodecyl sulphate. Equal amounts of protein were then loaded onto SDS-PAGE gels and transferred to PVDF membranes. Membranes were blocked and then probed with antibodies against SENP1, Bcl2 (both Santa Cruz); PARP and β-actin (both Cell Signalling Technologies, USA) overnight. Membranes were then washed and labelled with HRP-conjugated secondary antibodies for 1 h at room. Membranes were then washed in and protein bands were detected by chemiluminescence (Amersham, USA). Band densities were quantified using ImageJ analysis.

### 3.10. Statistics

Data are shown as the mean ± SD. Data were compared using a Student’s -test. P-values ≤0.05 were considered statistically significant. Data were analysed on GraphPad Prism 7 software.

## 4. Discussion

Recently, hADSCs have gained intense clinical interest due to their outstanding potential for a range of important human diseases. As for MSCs derived from other sources, hADSCs show stem cell-specific surface marker expression, but lack typical haematopoietic markers [24]. The main advantage of hADSCs over MSCs is their ability to be handled, harvested, and expanded through minimally invasive procedures. In addition, hADSCs are more stable in both genetical and morphological aspects showing higher rates of proliferation capacity and lower rates of cell senescence [25]. Despite these clear advantages, the use of hADSCs remains in its infancy.

SENP1, as a member of family of SENPs which remove SUMO from their substrates, play critical roles in the regulation of SUMOylation and deSUMOylation [26]. It has been shown that SUMOylation is a highly transient posttranslational protein modification that can influence cell cycle, cell proliferation, apoptosis, and cell differentiation [27]. To date, several reports have illustrated the essential roles of SENP1 in cancer research. It was previously demonstrated that the silencing of SENP1 inhibits the growth, migration, and survival of human glioma cells [28]. It was similarly reported that the Inhibition of SENP1 in neuroblastoma cells significantly suppressed their migration and invasion capacity [29]. SENP1 silencing also attenuated the growth and invasiveness of triple-negative breast cancer cells [30]. The culmination of these studies implicates SENP1 in the numerous important physiological and basic functions of cells.

Despite this knowledge the major functional roles of SENP1 in hADSCs have not been defined. In this study, we demonstrated that SENP1 significantly alters the functions of hADSCs through CRISPR/Cas9 mediated silencing. We further found that SENP1 silencing in hADSCs could inhibit the migration and proliferation of hADSCs, and the cell cycle was significantly arrested at the G0/G1 phase, which promoted apoptosis. These effects revealed new roles of SENP1 in hADSCs. Previous studies showed that the inhibition of SENP1 could reduce cell proliferation and induce apoptosis of multiple myeloma cells through the modulation of NF-κB signalling [31].

Also, and surprisingly, SENP1 did not change the morphology and MSC-related phenotypes of hADSCs. In our previous study, we found that mice harbouring an adipocyte-specific deletion of SENP1 developed glucose disorders due to dysregulated NF-kB signalling mediated regulation of NEMO. These data indicate a significant role of SENP1 in the regulation of cell proliferation, migration, and survival in hADSCs. Further studies are now required to understand the underlying mechanisms of hADSCs regulation.
